# Key Factors Shaping Successful Implementation of the Internet of Things (IoT) in Health Care: Qualitative Study

**DOI:** 10.2196/71546

**Published:** 2025-06-27

**Authors:** Klas Palm, Carl Kronlid, Marie Elf, Anders Brantnell

**Affiliations:** 1 Department for Industrial Engineering and Management, Uppsala University, Lägerhyddsvägen 1, Uppsala, 752 37, Sweden, 46 729999234; 2 School of Health and Welfare, Dalarna University, Falun, Sweden

**Keywords:** IoT, Internet of Things, implementation, health care, system, sociotechnical systems, case study

## Abstract

**Background:**

The utilization of the Internet of Things (IoT) can significantly enhance health care. However, successful implementation of IoT requires a holistic approach including factors beyond technology alone.

**Objective:**

This paper seeks to advance understanding of the factors influencing the successful implementation of IoT solutions in the health care sector, expanding beyond a purely technological focus.

**Methods:**

Using data from 22 semistructured interviews with a diverse group of stakeholders—including health care professionals, researchers, municipal and regional officials, and private companies—this study examines 5 leading IoT projects in Sweden.

**Results:**

Grounded in sociotechnical systems theory, the research identifies five critical subsystems impacting IoT implementation: (1) laws and regulations, which present challenges due to their complexity and misalignment with rapid technological advances; (2) organizational support, highlighting the essential commitment and resources from management to drive innovation; (3) user focus, emphasizing the importance of engaging end-users—such as patients and health care providers—in the design and implementation of IoT solutions; (4) resources, encompassing both financial investments and human capital needed for effective deployment; and (5) infrastructure, which addresses the technological foundations required to support IoT systems reliably.

**Conclusions:**

By shifting attention from adoption to the complexities of implementation, this study fills a critical gap in the literature, which has largely emphasized adoption and technical aspects over practical implementation challenges. The findings provide a nuanced understanding of the primary factors influencing IoT implementation in health care, illuminating both the challenges and potential avenues for successful integration. Ultimately, this research advances the sociotechnical systems theory and also offers valuable insights for managers and policymakers tasked with driving digital transformation in health care systems.

## Introduction

### Background

The Internet of Things (IoT) and technologies, such as artificial intelligence, big data, and cloud computing [1], can significantly enhance the quality of health care [2]. IoT is a network of intelligent sensing devices and physical objects that are digitally connected for the collection, monitoring, and control of health care data [3]. It enables tracking, identification, authentication, data collection, and sensing [4]. These features can lead to improved patient outcomes, increased efficiency, remote monitoring, and enhanced collaboration between health care providers [5]. They are essential to addressing rising demands, such as an aging population, an increase in long-term conditions, limited resources, and growing expectations for personalized health care [6].

However, in exploring IoT’s potential in health care, it is essential to distinguish between adoption and implementation. Adoption refers to the decision to use an innovation, while implementation involves the actual use of that innovation [7]. This distinction is often overlooked, as the 2 terms frequently used interchangeably or without clear differentiation [2,8-11]. IoT adoption and its influencing factors have been widely researched, typically focusing on technology [12-14], but also covering aspects related to individuals and the surrounding environment [15]. Adoption studies typically draw on frameworks such as the technology acceptance model (TAM) [16,17] and the Unified Theory of Acceptance and Use of Technology (UTAUT) [18,19].

A recent sociotechnical analysis of factors influencing IoT adoption in health care, based on a literature review and carried out by the Swedish researchers; Kronlid et al [20] identified a broad array of factors, 94 in total, that impact IoT adoption in health care. The comprehensive literature review by Kronlid et al serves as a starting point for this article, as it responds to previous calls for a deeper sociotechnical understanding of IoT adoption within health care and proposes a specialized sociotechnical systems framework tailored specifically to IoT adoption in health care settings. The review found no studies on the actual implementation of IoT in health care and only a few focusing on IoT adoption. Kronlid et al call for more rigorous empirical research on IoT adoption and implementation, grounded in frameworks that reflect sociotechnical realities.

Other previous research has shown that successful implementation requires consideration of factors beyond technology [21]. As early as 1951, Trist and Bamforth [22] emphasized the importance of social factors in realizing the full potential of technological innovations. Earlier research on implementing medical innovations shows that influencing factors extend beyond technical considerations to include managerial, clinical, financial, legal, and individual elements [23,24]. However, research exploring IoT implementation in health care through a sociotechnology lens remains scarce [20].

Rogers [7] defines adoption as “a decision to make full use of an innovation as the best course of action available,” emphasizing the importance of the decision-making process. Research on IoT adoption in health care began to gain traction around 2013, with a predominant focus on technological aspects [25]. Dantu et al [25] identified 5 main topic clusters for adoption: security and privacy, cloud and smart health, wireless network technologies, data, and applications. IoT solutions in health care rely heavily on the transfer of large volumes of data (ie, big data) and device-to-device communication, making high-speed internet a crucial requirement [26]. A review of studies on IoT in elderly care also highlighted bandwidth and network connectivity (eg, WiFi) as essential preconditions for IoT adoption [27]. In addition, cloud services play a significant role in IoT adoption, providing big data storage and access, often described as the “backbone” of IoT solutions [12]. Several studies have explored the benefits and risks associated with cloud services [13,14,28-31].

The literature addressing nontechnical aspects of IoT adoption in health care is relatively limited, and the lack of focus on the social dimensions of IoT adoption has been noted by several researchers (eg, [8,25]. In a review of IoT adoption in health care [15], categorizing the factors influencing IoT adoption into 5 groups: individual factors (eg, social influence), technological factors (eg, perceived ease of use), security factors (eg, trust), health factors (eg, perceived health risk), and environmental factors (eg, financial cost). Many of the included studies applied the TAM or the UTAUT1 and UTAUT2 [15].

An interesting finding is that Alraja [32] found that gender influences the intention to use IoT technology among Generation Y, but not among Generation X. Furthermore, concerns about privacy risks did not impact the decision to use IoT technology. Additionally, Huarng et al [33] developed a model for the adoption of health care wearable devices, identifying data privacy, perceived ease of use, and perceived usefulness as key influencing factors. The review by Kronlid et al [20], which found 94 factors influencing IoT adoption, was grouped into eight subthemes: (1) financial circumstances (eg, reimbursement), (2) infrastructure (eg, data management), (3) people (eg, attitudes and feelings), (4) procedures (eg, capability development), (5) regulatory frameworks (eg, security and privacy), (6) stakeholders (eg, commercialization challenges), (7) technology (eg, complexity), and (8) other factors (eg, conflicts of interest). Of these, 34 factors were related to technology and infrastructure, accounting for approximately 42% of the total. The categories people and regulatory frameworks each counted for around 20%. Thus, this highlights technology, infrastructure, people, and regulatory frameworks as the most important areas. Notably, many of these factors were identified from research in low- and middle-income countries [20], raising questions about their relevance in high-income settings.

### IoT Implementation in Health Care

Implementation occurs when an individual or organization actively begins using an innovation [7] and can be understood as “a deliberately initiated process, in which agents aim to bring into operation new or modified practices that are institutionally sanctioned and performed by themselves and other agents” [34]. A recent review did not find any studies specifically addressing IoT device implementation [20]. Moreover, distinctions between adoption and implementation are often blurred or unclear in the existing literature [20]. To gain insights into the range of nontechnical factors influencing IoT implementation, it is useful to consider findings from related fields, such as telemedicine, where technology integration in health care shares similarities with IoT [3]. In telemedicine research, the difference between adoption and implementation is more distinctly delineated; see, for example, Broens et al [35].

Telemedicine implementation is a well-established research area, with several reviews summarizing key findings. Broens et al [35] identified 5 primary factors influencing telemedicine implementation: technology (eg, quality), user acceptance (eg, attitudes), financial considerations (eg, reimbursement structure), organizational aspects (eg, work practices), and policy and legislation (eg, standardization). Scott Kruse et al [23] identified 5 common barriers to telemedicine implementation globally: technically challenged staff, resistance to change, cost, reimbursement issues, and demographic factors such as patient age and education. Recent studies validate these findings; for example, Khodadad-Saryazdi [24] emphasized that successful implementation requires adaptability in technology, strategy, and culture. Similarly, Cannavacciuolo et al [36] affirmed the 5 factors initially outlined by Broens et al [35]. Overall, successful implementation is a mix of technical and nontechnical factors.

Most previous empirical research on IoT implementation in health care is quantitative, using surveys to collect data [2,8,9,15] combined with adoption theories such as TAM or UTAUT. While useful, there are drawbacks that we have identified in previous research. The combination of quantitative methodologies and adoption theories limits the research, as it lacks the capacity to include unexpected aspects of IoT implementation. We thus see the need to broaden the literature both in terms of methodology and theory: methodologically, with more qualitative studies that have more flexibility to let the data guide the research, and, theoretically, to identify and make sense of, for example, organizational and contextual factors that can play a major role in the implementation of IoT.

In summary, research demonstrates the importance of considering many factors when implementing new health care technologies. This highlights the need for more empirical studies on IoT implementation that draw on a sociotechnical systems framework to account for the complex interplay of these factors.

### The Sociotechnical Systems Model

**Figure 1.**
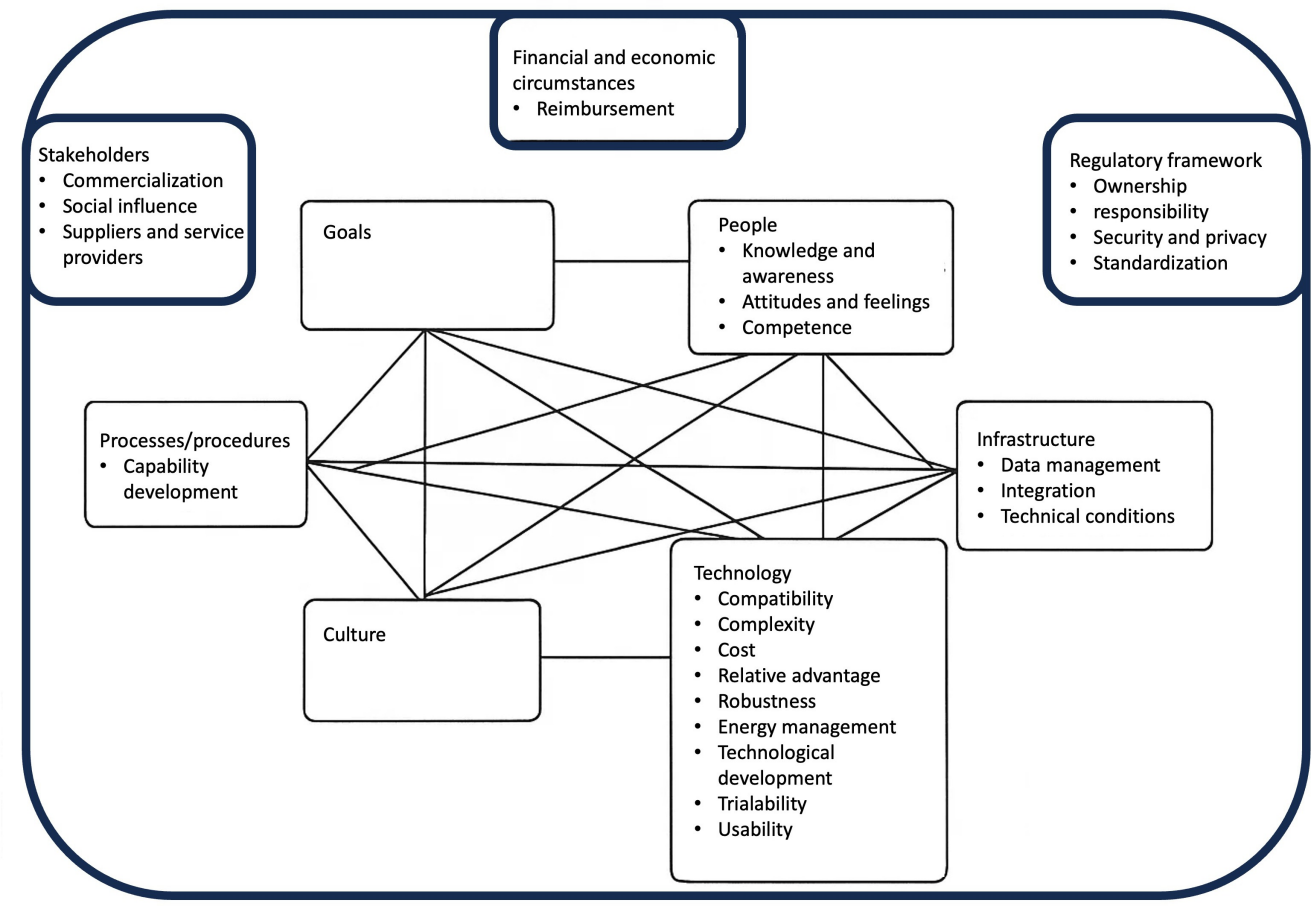
Sociotechnical systems model (adapted from Kronlid et al [20], which is published under Creative Commons Attribution 4.0 International License [39]).

This study builds upon the sociotechnical systems framework proposed by Kronlid et al [20], which categorized key factors influencing IoT adoption in health care settings (see Figure 1). The framework is informed by and aligns with implementation research in related fields, such as telemedicine [23,35], thus providing a solid foundation for analyzing the multifaceted challenges and enablers associated with IoT implementation in health care.

The sociotechnical systems model, grounded in the work of Leavitt [37], highlights that any system is composed of multiple, interdependent subsystems. Changes in one subsystem, such as technology, will invariably impact others, including organizational culture, infrastructure, or processes. A more modern version of this approach, developed by Davis et al [38], outlines 6 key subsystems within an organizational system: goals, technology, culture, people, infrastructure, and processes. These subsystems interact with an external environment that includes regulatory contexts, financial factors, and other stakeholders. While this model has proven useful in many areas, Kronlid et al [20] propose that specific modifications might be needed in health care. Their review showed that the “culture” and “goals” subsystems of the model have not been well studied in health care research.

This study investigates the key factors influencing IoT implementation in health care, based on empirical evidence from 5 state-of-the-art IoT projects in Sweden. These projects were pilot projects; the focus of the study was on the early stages of the implementation process.

We explored how various factors, particularly organizational and societal, contributed to successful implementation of IoT. Using a comparative case study approach, we analyzed the key mechanisms driving the integration of IoT in health care. Through this analysis, we also aimed to refine sociotechnical systems theory as it pertains to IoT implementation in healthcare.

We moved beyond traditional adoption studies that often rely on frameworks like UTAUT, which, while useful, do not capture the complexity of implementing new technology in practice. Instead, we applied the 7 subsystems proposed by Kronlid et al [20], tailored for health care but flexible in terms of specific influencing factors within each subsystem. Since their framework was developed from a limited number of adoption-focused studies, we did not predefine factors within each subsystem. Instead, we remained open to identifying new factors, allowing a more comprehensive understanding of IoT implementation in health care.

In this article, we aimed to deepen theoretical insights into the critical factors affecting the early phase of implementation. We identified factors influencing the first phases of IoT implementation in health care through the lens of sociotechnical systems theory. Accordingly, our research questions were: (1) What are the most important factors that influence the early phase of implementation of IoT in health care? (2) How can sociotechnical systems theory contribute to understanding IoT implementation in health care?

## Methods

### Study Design

The study uses a qualitative multiple-case approach [40], suitable for generating new insights and contributing to theory development. This approach is particularly useful for studying emerging areas such as IoT implementation in health care when an in-depth understanding of the phenomenon is needed [40,41]. Using multiple cases allows for replication logic where cases are compared with each other to create robust and reliable insights [41,42].

### Case Selection

To identify appropriate sources of information, we reviewed 72 state-of-the-art IoT development initiatives funded by a Swedish agency specializing in IoT solutions. By analyzing the applications and mid-term reports, we identified 20 initiatives directly related to IoT applications within health care. Among these, 5 initiatives were selected as relevant for studying implementation.

These 5 cases represent small-scale IoT pilots, each testing practical applicability in health care. The pilot nature of these projects, with varying numbers of end users (patients), provided insights into implementation factors under controlled conditions before any larger-scale deployment. We initially aimed to include full implementation cases; our review showed that none had reached that stage. Instead, these pilots served as the closest point to practical implementation available for study.

Notably, none of the used IoT technologies had undergone the public procurement process required for broader implementation within Sweden’s health and social care systems. Multimedia Appendix 1 provides an overview of these 5 cases.

### Data Collection

We started the data collection by first reviewing the existing documentation (eg, grant applications and follow-up reports) concerning each of the projects to map all partner organizations in each of the projects and identify the key players and the project managers leading the initiative. Then, we conducted semistructured interviews with the project managers in each project and regarded them as key informants [43]. We also asked the project managers to identify other relevant partner organizations that we should interview to obtain a broad picture [41]. We conducted 22 interviews altogether, which lasted between 22 and 57 minutes. We followed an interview guide that covered background questions, questions concerning the initiative, and possible factors influencing implementation. The interviews were recorded and transcribed. Table S2 in Multimedia Appendix 2 provides an overview of interviews.

### Data Analysis

The analysis of the transcribed interviews was conducted using an abductive method [44]. We started with an inductive systematic thematic analysis [45] to identify patterns grounded in the participants’ perspectives. These identified themes were then organized according to the 7 subsystems proposed by Kronlid et al [20], allowing us to relate the empirical findings to the theoretical framework.

The process involved the following 7 stages. First, following Braun and Clarke [45], we familiarized ourselves with the data by thoroughly reading and rereading the transcripts. This stage aimed to gain a comprehensive understanding of the content and context of the participants’ responses, noting initial ideas and recurring topics that emerged throughout the interviews. Second, we identified and generated initial codes from the transcribed interviews. This involved systematically coding segments of text that reflected factors influencing the implementation of IoT solutions in health care. Each segment was labelled with a code that encapsulated its essence. The coding process was flexible and data-driven, ensuring that the codes emerged directly from the participants’ responses rather than from preconceived notions. Two researchers coded the transcripts independently to capture diverse interpretations and perspectives, which were then discussed to reach consensus.

Third, we grouped the initial codes into broader themes. This involved examining the codes for patterns and relationships and identifying clusters that represented similar ideas or concepts. The entire research team collaborated to discuss and refine these potential themes, ensuring they accurately reflected the data and contributed to addressing the research questions. Fourth, once the initial themes were established, we engaged in a thorough review process to refine and validate them. This involved checking whether the themes accurately represented the coded data and aligned with the overall dataset. The researchers revisited the interview transcripts to ensure the themes were robust and reflective of the diversity of participant experiences. Any inconsistencies or gaps were addressed through discussions and iterative adjustments. Fifth, after finalizing the themes, we aligned them with the sociotechnical subsystems by Kronlid et al [20] to the extent possible. However, we used inductive labeling, which provided a more accurate reflection of the data. As a result, the findings are structured around five adapted subsystems drawn from Kronlid et al [20] framework: (1) regulatory framework, (2) organizational support, (3) user focus, (4) financial and economic circumstances, and (5) infrastructure. The remaining 2 subsystems in the original framework, “technology” and “stakeholders” [20], did not emerge from the data and are therefore not included in the findings.

Sixth, to identify the specific factors influencing implementation of IoT in health care, we revisited the data within each of the 5 subsystems. This allowed us to break down the data into distinct factors linked to each subsystem. For details, see Table S3 in Multimedia Appendix 2.

Seventh, we compared the subsystems and factors across cases, identifying similarities and differences [41]. Finally, we compiled the data into a coherent findings section, aiming to convey the rich insights derived from the analysis and highlighting how various factors influence the implementation of IoT solutions in health care.

### Ethical Considerations

The Swedish Ethics Review Authority made a rejection of the request for ethical review as they considered that ethical review was not necessary, which means that a favorable ethical decision was obtained (exemption number: Dnr 2022-01259-01). The data were collected through interviews, and the participants chose to agree to the interviews. The original consent covered secondary analysis without additional consent. Data were anonymized. No compensation was provided to participants.

## Results

Our findings show that factors within 5 subsystems influence implementation of IoT in health care.

### Regulatory Framework

Respondents from all but one of the cases we studied identified laws and regulations as major factors influencing the implementation process. In the IoT Older Adults Home Care project, challenges related to laws and regulations were identified early on, and the project was designed for straightforward implementation. The primary issue concerned informed consent from the older adults. The project targeted older adults, including individuals with neurocognitive disorders such as dementia; the implementation faced difficulties due to the inability of older adults to provide informed consent. In one participating municipality (Municipality A), this was taken into account when selecting participants, and no major issues arose. However, in another municipality (Municipality B), the decision was made to include older adults who could not provide informed consent, which led to a failed implementation in that municipality.

*But you could say that [Municipality B] actually failed due to issues with consent and so on. So, we made installations in [Municipality B], but it didn’t work out. Because they wanted to include a user group that couldn’t give their consent. Legally, it fell through because they couldn’t study the individuals they wanted to include in the project.*

[Respondent 11, IoT Older Adults Home Care & IoT Older Adults Care Home projects]

This issue, where those who might benefit the most from IoT technology are unable to provide informed consent, may stem from laws and regulations that have not kept pace with recent technological advancements. One respondent specifically mentioned that the laws and regulations are “outdated,” emphasizing how this discrepancy creates challenges from a user perspective.

*If you look at how laws are written and changed, the time we’ve had digital tools is very short compared to how long we’ve had the Patient Data Act. We have several... quite... or very strict laws that we work under when working in healthcare, and they are outdated when it comes to... […] Patient safety and user-friendliness don’t really go hand in hand. So that’s one of the major obstacles when talking about [IoT] implementation.*

[Respondent 16, IoT CF project]

Some strategies were used to navigate existing laws and regulations. This was particularly evident in the IoT CF (cystic fibrosis) project, where creative approaches were used to deliver IoT services to patients and collaborate with companies. One approach involved “hiding behind studies,” using ongoing research studies as a way to test and implement IoT technology. Research studies have less demanding regulatory requirements, so hospitals could not object to the activities conducted within them. In addition, the involved companies were not permitted to integrate with specific health care systems. As a workaround, they used the diabetes registry, which operates under different rules.

*And we hid behind studies. Studies have much lighter requirements, and you can test all the technology. So, it turned out that we started new studies, and we have several ongoing studies because the hospital couldn’t say anything. That way, we could hide behind them with the technology. […] My actual research is in something else. This is not my research area, even though I conduct studies, but it’s just to hide behind them. As we said, it makes it easier to get around these technical...*

[Respondent 15, IoT CF project]

In the IoT LTC (long-term condition) project, respondents emphasized the challenges of handling and securing data in compliance with relevant regulations. Ensuring that data was collected, stored, and used according to legal standards was crucial for maintaining both trust and legality. Meeting certification standards was necessary for the project’s legitimacy and acceptance, but obtaining these certifications was a complex process that required careful attention to detail. In the IoT Older Adults Care Home project, there was uncertainty about how the technology should be classified and, consequently, which laws and regulations would apply. The technology captured a clear image of the older adult, which was later blurred on a server. Since a clear image was initially captured, the municipality determined that the camera surveillance law was applicable, leading to the shutdown of the technology testing and a change in the project’s direction. After extensive discussions with the municipality’s legal team, it was concluded that they should have used a camera instead of a sensor. Had the camera blurred the image immediately, or if the server had been located within the same facility, a different decision might have been reached.

*So, we use motion sensors and... yes, employ a different interpretation pattern around it or under the radar, you could say that the data you collect about the person is essentially the same. But in our case, it’s the camera law that applies.*

[Respondent 14, IoT Older Adults Care Home project]

In the IoT CF, IoT Bedwetting, and IoT LTC projects, the public procurement process was identified as a significant challenge. The difficulty lay in ensuring that procurement was conducted legally while still allowing for an innovative approach, which was critical to the success of these projects. The IoT CF project described the procurement process as very slow, with the risk that the product would become outdated during the procurement process. In the IoT LTC project, respondents highlighted how procurement remains a challenging issue, with the belief that the continued implementation of IoT solutions almost always stalls once it reaches the procurement stage. Several respondents suggested that this issue is linked to lawyers’ reluctance to test new procurement techniques, driven by a fear of potential legal risks.

*Then you get into the procurement issues concerning regions and municipalities, and it becomes very, very challenging in many ways. Not least, there is a fear from the actors in, for example, this project. Are they allowed to participate in a procurement, or do they know too much now about the customers in different ways, making them disqualified from the procurement? That risk always exists. So, this procurement step is very, very difficult in the next stage.*

[Respondent 21, IoT LTC project]

### Organizational Support

Respondents from all the cases we studied emphasized the importance of organizational support in creating an enabling environment for IoT implementation. In the 2 projects, IoT Older Adults Home Care and IoT Older Adults Care Home, both initiated by the same municipality, it was clear that the municipal management had made a firm decision to support digitalization initiatives. This decision, coupled with its effective communication, was considered highly beneficial to the success of these 2 projects. The IoT project manager received strong backing from various levels of organizational leadership. The municipality itself was “very focused on introducing various types of technology within the care services” (Respondent 13, IoT Older Adults Home Care & IoT Older Adults Care Home projects). In the Older Adults Care Home project, managers and staff at the facility where the technology was being tested were particularly enthusiastic and supportive.

*The housing manager, department manager, and many within the organization are truly passionate about this and are very driven, visionaries, and willing to take risks to make it as good as possible for the individuals. So, we really see that as a success factor in the project.*

[Respondent 12, IoT Older Adults Home Care & IoT Older Adults Care Home projects]

Both the IoT CF and IoT LTC projects also highlighted the importance of support functions, such as IT services, administrative assistance, and training, in ensuring successful implementation. The IoT CF project further illustrated the challenges of advancing IoT implementation when facing resistance from organizational stakeholders and a lack of support and resources necessary to establish a more permanent solution. In this case, the IoT project manager encountered significant pushback from colleagues, management, and support functions like the IT department.

*There are many parts of the IT structure, and we had everyone against us, I must say. Everyone thought it was good, but many thought it was wrong [some technical aspects were deemed illegal by the IT department]. Many didn’t think we should do what we did, but then [if we did it] be held accountable for it.*

[Respondent 15, IoT CF project]

Several respondents across all cases noted that the level of prioritization within the organization directly impacted a project’s progress and success. A common observation was that organizational priorities and decisions outside the project’s scope often had a negative influence. For example, in the IoT Older Adults Home Care project, one respondent raised the influence of the political planning: “There’s a political agenda that usually follows a 4-year cycle, a planning horizon for operations. So, there are many other factors that govern what actually happens [in projects like this]” (Respondent 7, IoT Older Adults Home Care project). Several respondents also highlighted the difficulty that lower-priority projects had in gaining momentum.

*The third is getting priority in the regions to obtain staff and resources to assist in the projects is almost impossible, so even if the staff wants to, it is very difficult for them to, well, take care of these projects.*

[Respondent 18, IoT LTC project]

Additionally, one project raised an aspect crucial to its success but not mentioned by any other project: organizational readiness for implementation. Respondents from the IoT Older Adults Home Care project emphasized that the level of knowledge regarding implementation varied significantly among managers at different organizational levels, influencing the feasibility of implementing the IoT solution.

*There is a large variation in maturity levels [knowledge of implementation] among our operational managers. And when I say operational managers, I mean everyone from those at individual care facilities, group homes, to the highest leadership. They don’t have a unified understanding [of what to do and how to do it]; it’s quite diverse.*

[Respondent 6, IoT Older Adults Home Care project]

### User Focus

Another major success factor identified in all the cases we studied was having a strong user focus, which encompassed both patients and health care staff.

*It originates from the staff and patients from the start, and they have been involved throughout and have had a say in how it should look [like], how the tool should look [like], what functionality we should have, and what the priorities should be.*

[Respondent 21, IoT LTC project]

In the IoT LTC project, it became clear that involving users (ie, health care staff) not only helped the service developer improve the usability of their service but also increased the likelihood of successful implementation. Respondents noted that while the service developer initially had a vision for how the service should function, there was often a disconnect between the engineers’ perspective and how patients, relatives, and health care staff perceived the service. In the IoT CF project, the team had initial ideas about the effects and benefits the technology would bring to patients, but additional benefits were identified by both patients and their relatives.

*So, we made the technology available to everyone, instead of making decisions like “you think,” “we need,” or similar. And we saw quite clearly that it’s very difficult to guess who will benefit from the technology. [...] I think that [involving patients] was a success factor because we said, “This doesn’t mean it should replace one or the other,” but we let our patients choose instead of healthcare professionals making the choice.*

[Respondent 15, IoT CF project]

In addition to involving immediate users (ie, caregivers and patients), it is also essential to include a broader range of users, as demonstrated in the IoT CF project. This project engaged health care staff both directly and indirectly connected to patient care, such as medical secretaries, nurses, dietitians, psychologists, physiotherapists, and counsellors. This extensive involvement was highlighted as a key success factor. However, some respondents also pointed out the complexities that IoT technology introduces to caregiving, noting that health care staff may feel that the more technology is introduced, the further they move away from their core role as caregivers. This creates a paradox, as many choose to work in health care precisely to be close to the patient.

*We have gained a lot of insight into the technology frustration that exists [i.e, the complex role of technology in caregiving] and that, as it is today, they [the health care staff] feel they are moving further away from their caregiver role than they would like [to].*

[Respondent 12, IoT Older Adults Home Care project]

A match between user instructions and user capabilities is also crucial in IoT implementation projects. Both the IoT CF and IoT Older Adults Home Care projects emphasize the importance of clear communication with users and adjusting the complexity of instructions to suit their abilities. Additionally, one project (IoT Bedwetting) highlighted the role of national guidelines—care guidelines from authorities that outline best practices in different clinical areas—in the success of IoT implementation. Respondents from the IoT Bedwetting project suggested that if national guidelines recommend IoT solutions, this could become a critical factor in achieving successful outcomes.

### Financial and Economic Circumstances

Securing sufficient resources for implementing IoT solutions was highlighted as a challenge in several interviews across the IoT LTC*,* IoT CF*,* and IoT Older Adults Home Care projects. These resources primarily include financial support. Shorter projects are often insufficient to drive meaningful change, as it takes considerable time—and therefore money—to alter established practices. There is often ambiguity around who should bear the costs for the necessary time, resources, and ongoing maintenance of the services.

*No one wants to pay. Everyone thinks someone else should pay. But now I have no... But now I’m a little... Now we’re shutting down if the hospital doesn’t step in and pay. So I’ve set some demands. And everyone thinks this group is good and should continue, but it’s... everyone thinks someone else should pay.*

[Respondent 15, IoT CF project]

### Infrastructure

Respondents from 3 cases emphasized that infrastructure is crucial for successful IoT implementation. They noted that the process was often hindered by either insufficient or unstable WIFI connections, as well as concerns over the use of cloud services. In the IoT Older Adults Care Home project, for example, the IoT solution had already been procured by the region for other applications. However, when installation began, it became clear that WIFI coverage was inadequate to get the technology fully operational.

*Exactly, it’s another project that is implementing the equipment we intend to use in this project. In that project, I perceive that the staff has some resistance because there have been infrastructure issues that have prevented it from working as expected. For example, the WIFI hasn’t had the coverage they thought it would, and when the equipment lost network connectivity, it resulted in false alarms, in reality.*

[Respondent 12, IoT Older Adults Home Care & IoT Older Adults Care Home projects]

In the IoT Older Adults Home Care project, WIFI access was also a significant issue. Here, older clients were responsible for their own WIFI, complicating the service provider’s efforts to install the necessary infrastructure. In the IoT Bedwetting project, concerns about the security of cloud services also created challenges.

*...I have been in contact with [the region], and there the IT department flatly said no, that “we cannot agree to this because we do not consider cloud services to be secure.” So, that... those are the things I think are the problem, that there are many smart technical solutions, but in many cases, the healthcare system is not quite ready to embrace them.*

[Respondent 4, IoT Bedwetting project]

In the IoT Older Adults Home Care project, stakeholders such as the IT department seemed unaware of local cloud solutions and declined to consider them without further investigation. In contrast, the IoT Older Adults Care Home project successfully addressed potential security concerns by contracting with a local cloud provider.

In addition, communication tools play a critical role in supporting implementation. Our findings suggest that when communication tools are not properly aligned with the IoT solution, they can obstruct the implementation process.

*Then there are these contextual factors. For example, the staff currently use fairly old phones to receive their alarms, and this means that it takes quite a while... Every time an alarm comes in, it takes quite a long time for them to access their phones. They need to enter their code every time, open an app, and then turn off the alarm. And it takes quite a long time.*

[Respondent 12, IoT Older Adults Home Care & IoT Older Adults Care Home projects]

Another important aspect of communication tools is the use of web-based meetings. Our findings indicate that well-functioning web-based meetings can greatly facilitate implementation. In the IoT CF project, secure web-based meetings were essential for delivering the service. These meetings were facilitated centrally through a national service called “1177,” the primary digital platform for health care services in Sweden.

## Discussion

### Principal Findings

This study investigates the key factors influencing IoT implementation in health care by examining 5 state-of-the-art IoT projects in Sweden. It addresses the pressing need for more empirical studies on IoT implementation [20], as well as the need for an approach that goes beyond purely technical factors, focusing instead on a broader range of influences within organizational and social contexts [8,25].

**Figure 2.**
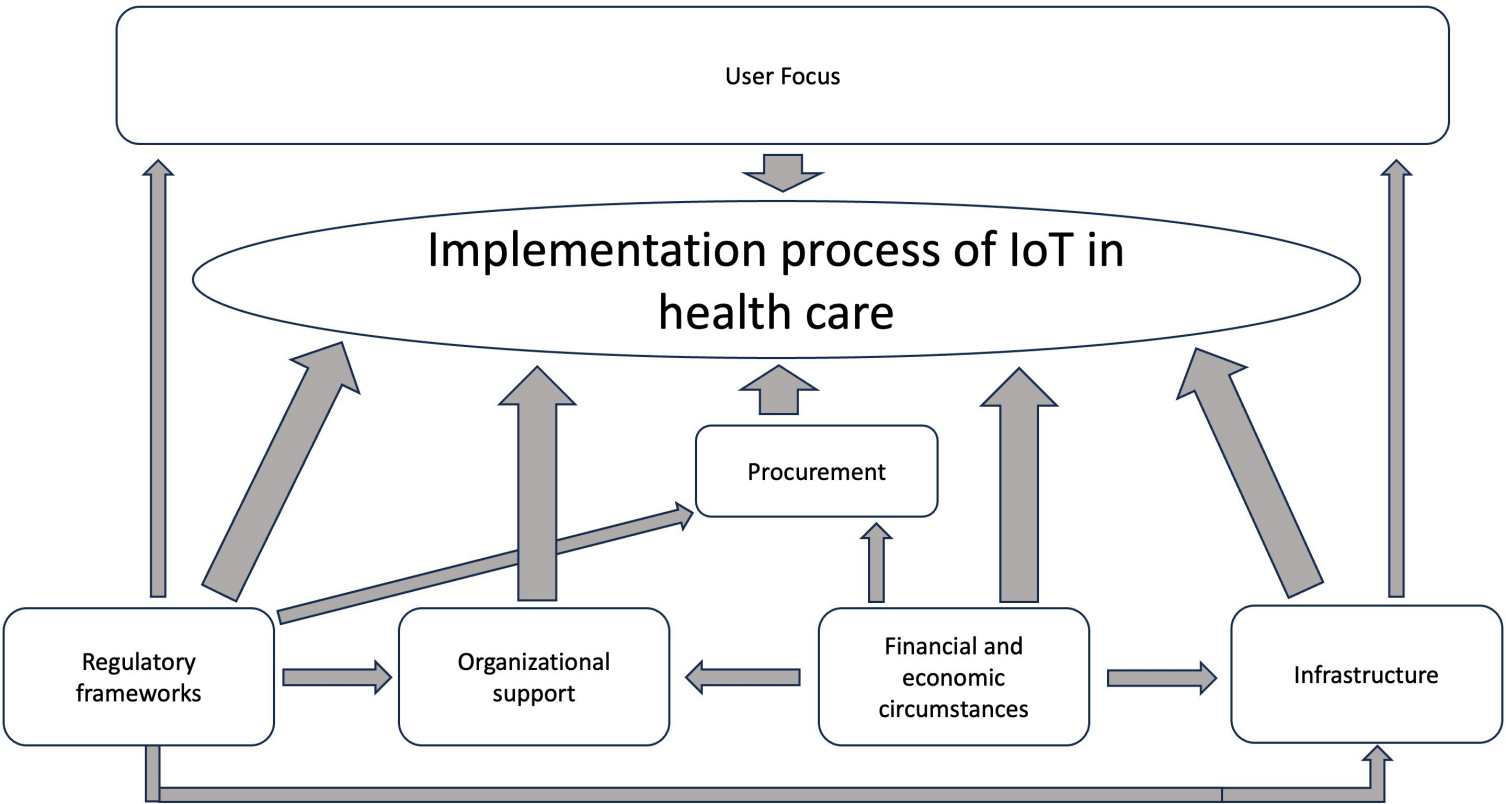
The most important factors that influence the implementation process of Internet of Things (IoT) in health care. The broader arrows represent a direct impact on the IoT implementation process in health care, while the narrower arrows represent an indirect impact—from one factor to another factor that in turn affects the ability to implement IoT in health care.

Figure 2 illustrates our theoretical contributions and proposes a sociotechnical systems model tailored to IoT implementation in health care. It is difficult to determine the exact causality between the different factors, but based on the interviews, Figure 2 illustrates our interpretation of the main relationships between the factors.

Our research contributes to the growing discourse on IoT in health care, which has predominantly focused on the adoption phase [5,15,25]. While prior studies emphasize technical factors as primary considerations during adoption [25,26], our findings underscore the importance of organizational and societal factors in the implementation phase. Through our study, we advance sociotechnical systems theory by demonstrating how its components interact in real-world health care settings, particularly in the context of IoT. Our findings reveal complex dynamics between technological potential and social structures, identifying key gaps, tensions, and interdependencies that impact implementation. Below, we discuss 5 primary ways in which our study contributes to applying sociotechnical systems theory to health care IoT implementation.

First, by applying the Kronlid et al [20] model, we identify specific refinements to the sociotechnical systems model necessary for the health care sector. Of the 4 core factors—people, infrastructure, technology, and processes or procedures [20]—our findings support 3. We relabel “people” as “user focus” and “processes or procedures” as “organizational support” to better reflect its relevance in health care. Infrastructure remains unchanged. The subsystem, “technology,” was not observed in the 5 cases we studied.

Second, our study confirms 2 of the 3 external factors in the sociotechnical systems model, financial circumstances and regulatory frameworks [20], but did not observe evidence for the third factor*,* stakeholders. More importantly, we suggest that financial circumstances and regulatory frameworks should be viewed as internal organizational factors because of their interconnectedness with other internal elements, particularly organizational support. For example, financial resources significantly influence organizational support: when funding is adequate, organizations can prioritize IoT projects, allocate resources, and provide leadership backing. Conversely, limited funding can restrict support and hinder even promising projects. Regulatory frameworks similarly shape user engagement by setting boundaries around privacy and consent, which are critical in health care. Regulations that protect vulnerable users can inadvertently restrict participation in IoT projects [46]. Thus, we propose that the sociotechnical systems model should reposition these factors as internal dynamics within health care IoT implementations.

Further research could examine whether the remaining subsystems from Kronlid et al [20], technology and stakeholders, are relevant to IoT implementation in health care beyond Sweden. Regarding the technology subsystem, the affordability and technical challenges of IoT solutions may vary across countries. In low-resource settings, such as parts of Africa, the high costs of IoT devices, infrastructure, and maintenance can hinder adoption. In contrast, high-income countries like the United States and Germany may face greater challenges in integrating IoT into existing health care systems, requiring significant investment in interoperability and cybersecurity.

As for the stakeholders’ subsystem, the commercialization of IoT solutions is shaped by regulations, market demand, and funding availability. In countries with strict health care regulations (eg, the European Union [EU] under General Data Protection Regulation), compliance costs can make it difficult for startups to scale their solutions. Meanwhile, in developing markets, a lack of investor confidence and infrastructure limitations may slow commercialization, even when there is strong health care demand.

Third, our findings highlight the essential role of health care workers’ attitudes and patient involvement in harmonizing social and technical elements of IoT adoption. The observed frustration among health care staff with certain technologies supports the sociotechnical systems theory assertion that social environments such as attitudes, values, and identities strongly influence technology use [47]. This insight suggests that future adaptations of sociotechnical systems theory could benefit from explicitly addressing the emotional and identity-related impacts of technology, particularly in health care, where professional identity often ties closely to user autonomy and caregiving roles.

Fourth, our study reveals that regulations often lag behind technological advances, creating obstacles to IoT implementation. Rather than facilitating adoption, regulatory complexity can become a barrier, consistent with previous findings on medical device regulations [48]. Health care’s rule-driven culture fosters a cautious approach, limiting the adoption of emerging technologies [49]. Our findings further indicate that existing procurement structures can impede innovation, as traditional processes stifle creative thinking necessary for IoT implementation. Although innovation-based procurement strategies are promoted in the EU [50], none of the cases we examined identified these as practical solutions. To enable effective IoT adoption, managers must understand legal and regulatory constraints while collaborating with legal teams, a rarely studied area in management research [51]. Without this collaboration, legal departments may adopt overly cautious stances, leading to project rejections, while support departments may resist due to anticipated increases in workload. In one case, the organization strategically managed data by using local servers to avoid EU data-sharing restrictions [52,53], demonstrating that creative solutions can help navigate regulatory challenges. The respondents mentioned that they were hiding behind academic studies, ie, using ongoing research studies as a way to both test and implement IoT technology. It indicates a certain level of frustration regarding the lack of flexibility in the organizational structures.

Finally, the technical complexity of IoT systems, coupled with the need for simplicity in user interfaces, underscores the principle that technology and human systems must evolve together [54,55]. While IoT technologies provide advanced capabilities, their complexity can alienate users, particularly in health care, where ease of use is critical. This observation suggests a vital revision for sociotechnical systems theory: usability should be prioritized as a co-evolutionary requirement within sociotechnical design, not as an afterthought. This aligns with theories of sociomateriality, supporting the idea that intuitive, user-friendly design is essential in fields with high cognitive and emotional demands [54]. Thus, even if our study did not specifically address user engagement strategies tailored to vulnerable groups, such as individuals with cognitive impairments or mobility limitations, we recognize this as critical for future research. Practical methods such as participatory design and co-creation techniques should be used to ensure that IoT solutions are inclusive and accessible [56]. A stronger focus on user experience design will enhance the relevance, equity, and effectiveness of implementation efforts in health care.

### Practical Implications

This study reveals several practical implications for managers involved in health care IoT implementation, highlighting the importance of collaboration, financial strategy, training, and regulatory awareness. First, close engagement with legal departments is crucial. Developing a collaborative relationship with legal teams allows managers to address complex regulatory concerns upfront, avoiding overly conservative stances that could stifle innovation. To facilitate smoother project approvals, it may also be beneficial to provide legal teams with training specific to IoT technologies, helping them understand the unique aspects of these projects. Second, demonstrating the economic benefits of IoT initiatives is equally essential. Managers can strengthen organizational support by conducting detailed cost-benefit analyses that clearly illustrate potential financial gains. This approach not only helps in securing initial funding but also promotes a culture that values and supports innovative solutions. Third, investing in comprehensive training programs is also vital for successful implementation. By providing targeted training across all staff levels, including frontline workers, support departments, and management, managers can build the technical competencies necessary for IoT integration. Moreover, fostering cross-functional collaboration by forming interdisciplinary teams encourages the sharing of expertise, which can lead to more effective problem-solving and innovation.

Fourth, ensuring that patients, health care providers, and other stakeholders are involved in the implementation process can significantly enhance the relevance and acceptance of IoT solutions. Addressing the needs and concerns of these users from the outset ensures that solutions are practical and user-centered. Developing clear protocols for managing informed consent is particularly important when technologies involve sensitive components like sensors or cameras. Special attention to accessibility, especially for patients with cognitive disabilities, further reinforces the inclusivity of the implementation. Finally, engaging with policymakers to advocate for flexible and supportive regulatory frameworks is crucial. By working together, managers and policymakers can create a balanced approach that safeguards patient safety and privacy while fostering a regulatory environment that encourages digital innovation in health care. This proactive stance helps to create a foundation for sustainable IoT implementation, aligning technological advancement with patient care priorities and organizational objectives.

### Limitations and Further Research

Our study has 2 main limitations. First, it examines only 5 IoT pilot implementation cases, which, while allowing for a deep exploration of IoT implementation factors, limits the generalizability of our findings. The small sample size may mean that our conclusions are less applicable to other health care settings, particularly those outside Sweden. However, we believe that the results may very well be relevant for countries with similar demographics and systems for social and health care. Expanding future research to include a broader and more varied set of cases across different regions or countries would help to enhance generalizability. However, as shown by our sampling process, identifying numerous relevant IoT cases can be challenging. Second, each case we studied was in the pilot implementation stage, with none yet reaching full-scale public procurement or integration into Sweden’s health care system. This limitation restricts our insights into the long-term challenges and sustainability of IoT implementation in health care. Consequently, our findings do not fully capture the issues and opportunities associated with later stages of implementation, such as public procurement, scaling, and long-term use.

Building on these limitations, several avenues for further research emerge. One area needing exploration is the adaptation of health care laws and regulations to better support IoT implementation. Future studies might investigate how regulatory frameworks and procurement models, such as innovation-based procurement, could be refined to foster IoT implementation. Research could also focus on best practices for aligning health care regulations with the rapid advancements in IoT technology. Future research could also aim to identify actionable solutions to the challenges that have been identified.

Since this study is based on pilot projects, there is also a need for longitudinal research that extends beyond the pilot phase. Examining the full-scale implementation of IoT solutions would provide insights into additional challenges and opportunities, including public procurement, solution scalability, and sustained use over time. Further, expanding the scope to different regions or countries would enable comparative studies to assess whether factors influencing IoT implementation are universally relevant or specific to certain national or health care system contexts.

Another critical research direction involves deepening our understanding of effective user involvement in IoT design and implementation. Future studies could explore best practices for managing informed consent, especially for vulnerable populations, like individuals with cognitive disabilities. These studies could help shape policies that ensure the full benefits of IoT technologies reach all user groups, reinforcing the patient-centered approach essential to health care innovation.

### Conclusions

In conclusion, this study explored 5 IoT pilot implementation projects in Sweden, shedding light on key factors that influence the deployment of IoT solutions in health care. By responding to the demand for empirical research on IoT implementation, our findings contribute to an understanding of the practical challenges and organizational dynamics involved, expanding beyond the adoption-focused literature. While IoT has the potential to significantly enhance health care delivery, several barriers remain, especially concerning regulatory complexity and rigid legal frameworks. These findings underscore the importance of ongoing collaboration between managers and legal teams to prevent regulatory constraints from hindering innovation. Our study also highlights the necessity of strong leadership in championing IoT projects within health care organizations. Managers play a pivotal role in clearly communicating the economic value of IoT through cost-benefit analyses and fostering a collaborative, cross-functional culture to tackle implementation hurdles. Adopting a user-centered approach—one that involves health care providers, patients, and families throughout the process—emerges as essential for achieving broad acceptance and success of IoT solutions. Additionally, managing informed consent, particularly for vulnerable populations such as individuals with cognitive disabilities, is crucial for the ethical deployment of these technologies. Overall, our study underscores the need for thoughtful strategies that prioritize regulatory alignment, economic rationale, and user involvement to realize the full potential of IoT in health care.

## Supplementary material

10.2196/71546 Multimedia Appendix 1 An overview of the 5 cases.

10.2196/71546 Multimedia Appendix 2 An overview of interviews and an overview of subsystems and specific factors influencing implementing IoT solutions in health care. IoT: Internet of Things.

## Abbreviations

CF: cystic fibrosis
EU: European Union
IoT: Internet of Things
LTC: long-term condition
TAM: technology acceptance model
UTAUT: Unified Theory of Acceptance and Use of Technology
