# Supplementary material for: Key Factors Shaping Successful Implementation of the Internet of Things (IoT) in Health Care: Qualitative Study
Source: JMIR Hum Factors. 2025 Jun 27;12:e71546. doi: 10.2196/71546 (PMC12288103; doi:10.2196/71546)
Supplement: Multimedia Appendix 1 [file humanfactors-v12-e71546-s001.docx]

**Appendix A**

Table 1. Overview of cases

| **Cases** | **IoT Long-Term Condition (LTC)** | **IoT Cystic Fibrosis** (**CF)** | **IoT Bedwetting** | **IoT Older Adults Home Care** | **IoT Older Adults Care Home** |
| --- | --- | --- | --- | --- | --- |
| Area | LTC | CF | Bedwetting | Older Adults home care | Older Adults care home |
| Funding period | 2021-2023 | 2021-2023 | 2021-2023 | 2017-2020 | 2021-2023 |
| Budget | 2.0 MSEK | 5.9 MSEK | 5.7 MSEK | 27.2 MSEK | 19.7 MSEK |
| Solution | Smartwatch and platform service | Home spirometry | IoT-based enuresis alarm | IoT sensors for home care | IoT sensors for Older Adults care homes |
| Target population | Kidney transplant patients | Children with CF | Children with bedwetting issues | Older Adults living at home | Older Adults living in care homes |
| Partners involved | 5 organizations:  Industry (n=2), healthcare (n=1), academia (n=1), research institute (n=1) | 6 organizations: Industry (n=2), healthcare (n=4) | 5 organizations:  Industry (n=1, healthcare (n=3), academia (n=1) | 16 organizations: Industry (n=9), healthcare (n=3), academia (n=3), research institute (n=1) | 5 organizations: Industry (n=1), healthcare (n=2), academia (n=2) |

Case descriptions

IoT Long-Term Condition (LTC)

“IoT LTC” was a collaborative initiative involving a university, two private companies, two municipalities, and a research institute. The project focused on supporting self-care and treatment compliance for patients with long-term condition kidney disease through an IoT tool. The solution aimed to assist in monitoring and reinforcing self-care behaviors, offering reminders, feedback, and training for sustained motivation and quality in self-care practices. This tool enabled healthcare providers to prioritize and allocate resources effectively by calling patients for in-person visits only when necessary. By maintaining close follow-up, the project sought to ensure that healthcare resources were utilized optimally, aligning with patient needs and supporting ongoing motivation for self-care.

IoT Cystic Fibrosis (CF)

“IoT CF” was developed in collaboration with four healthcare regions and a patient advocacy organization, focusing on improving the quality of life for children with Cystic Fibrosis (CF). This project introduced virtual care with remote monitoring of lung function and other health indicators at CF centers across Sweden. Through this IoT solution, about 50% of hospital visits were expected to transition to virtual consultations, allowing patients to conduct routine health assessments at home instead of at the hospital. The system aimed to create a more adaptable and family-centered care model while enabling effective resource utilization by reducing the need for in-person hospital visits.

IoT Bedwetting

The "IoT Bedwetting" project was a collaboration between a healthcare region and a private company focused on enhancing the treatment of enuresis (bedwetting). Traditional treatments involve using an alarm that sounds when bedwetting is detected, which can be effective but is often distressing for children and their families. The project aimed to develop an IoT solution that could eliminate the need for such disruptive alarms while improving treatment success rates from 50% to 80–90%. The IoT system was designed to monitor and manage bedwetting in a way that is more comfortable and less intrusive, providing tailored insights to help children and their families understand and manage the condition. By focusing on a personalized treatment approach, the project intended to reduce the stress associated with enuresis treatment, enabling a more compassionate and supportive experience for both children and their families.

IoT Older Adults Home Care

This project was a collaboration among three municipalities, ten companies, and three universities. The goal was to optimize elder care services by incorporating new IoT devices into existing municipal systems, allowing data from electricity and water meters and motion sensors to inform care decisions. These data sources helped develop profiles of daily activities for residents, enabling caregivers to detect any deviations that might indicate the need for intervention. By integrating various data points into a cohesive user profile, the system aimed to streamline care management for older adults while improving their quality of life through responsive and individualized support.

IoT Older Adults Care Home

"IoT Older Adults Care Home" was a follow-up to the "IoT Older Adults Home Care" project, involving collaboration among a municipality, a county, and three universities. This project focused on enhancing security and quality of life for nursing home residents through IoT solutions and AI-based analysis of life data. By tracking behavior patterns over time, the project aimed to identify trends that could indicate health risks and support more targeted, person-centered interventions. This system sought to improve residents' well-being by enabling timely responses to emerging health needs and providing objective data to support ongoing care and treatment decisions.
