# Supplementary material for: Key Factors Shaping Successful Implementation of the Internet of Things (IoT) in Health Care: Qualitative Study
Source: JMIR Hum Factors. 2025 Jun 27;12:e71546. doi: 10.2196/71546 (PMC12288103; doi:10.2196/71546)
Supplement: Multimedia Appendix 2 [file humanfactors-v12-e71546-s002.docx]

**Appendix B**

Table 2. Overview of interviews

| **Respondent** | **Project** | **Actor** | **Duration** |
| --- | --- | --- | --- |
| 1 | IoT Bedwetting | University and healthcare organization | 40 min |
| 2 | IoT Bedwetting | Private company | 38 min |
| 3 | IoT Bedwetting | Healthcare organization | 37 min |
| 4 | IoT Bedwetting | Healthcare organization | 33 min |
| 5 | IoT Older Adults Home Care | University | 36 min |
| 6 | IoT Older Adults Home Care | Municipality | 30 min |
| 7 | IoT Older Adults Home Care | Municipality | 22 min |
| 8 | IoT Older Adults Home Care | Governmental research institute | 43 min |
| 9 | IoT Older Adults Home Care | Private company | 32 min |
| 10 | IoT Older Adults Home Care | Company | 53 min |
| 11 | IoT Older Adults Home Care & IoT Older Adults Care Home | University | 57 min |
| 12 | IoT Older Adults Home Care & IoT Older Adults Care Home | Municipality | 41 min |
| 13 | IoT Older Adults Home Care & IoT Older Adults Care Home | University | 51 min |
| 14 | IoT Older Adults Care Home | Private company | 28 min |
| 15 | IoT CF | University and healthcare organization | 55 min |
| 16 | IoT CF | Healthcare organization | 44 min |
| 17 | IoT CF | Healthcare organization | 28 min |
| 18 | IoT LTC | Private company | 47 min |
| 19 | IoT LTC | Private company | 27 min |
| 20 | IoT LTC | Region | 42 min |
| 21 | IoT LTC | Governmental research institute | 37 min |
| 22 | IoT LTC | Private company | 29 min |

Table 3. Subsystems and specific factors influencing implementing IoT solutions in healthcare

| **Subsystems** | **Factors** |
| --- | --- |
| Regulatory framework | Informed consent |
|  | Outdated laws and regulations |
|  | Certification and classification |
|  | Public procurement |
| Organizational support | Management support |
|  | Support functions |
|  | Prioritization in the organization |
|  | Organizational readiness for implementation |
| User focus | User needs |
|  | User involvement |
|  | Role of technology in caregiving |
|  | Match between user instructions and user capabilities |
|  | National guidelines |
| Financial and economic circumstances | Financial means |
| Infrastructure | WIFI |
|  | Cloud |
|  | Communication tools |
